# HRProfiler Detects Homologous Recombination Deficiency in Breast and Ovarian Cancers Using Whole-Genome and Whole-Exome Sequencing Data

**DOI:** 10.1158/0008-5472.CAN-24-2639

**Published:** 2025-05-06

**Authors:** Ammal Abbasi, Christopher D. Steele, Erik N. Bergstrom, Azhar Khandekar, Akanksha Farswan, Rana R. McKay, Nischalan Pillay, Ludmil B. Alexandrov

**Affiliations:** 1 Department of Cellular and Molecular Medicine, University of California San Diego, San Diego, California.; 2 Department of Bioengineering, University of California San Diego, San Diego, California.; 3 Moores Cancer Center, University of California San Diego, San Diego, California.; 4 Bioinformatics and Systems Biology Graduate Program, University of California San Diego, San Diego, California.; 5 Sanford Stem Cell Institute, University of California San Diego, San Diego, California.; 6 Research Department of Pathology, Cancer Institute, University College London, London, United Kingdom.; 7 Department of Cellular and Molecular Pathology, Royal National Orthopaedic Hospital NHS Trust, Stanmore, United Kingdom.

## Abstract

Breast and ovarian cancers harboring homologous recombination deficiency (HRD) are sensitive to PARP inhibitors and platinum chemotherapy. Conventionally, detecting HRD involves screening for defects in *BRCA1*, *BRCA2*, and other relevant genes. Recent analyses have shown that HRD cancers exhibit characteristic mutational signatures due to the activities of HRD-associated mutational processes. At least three machine learning tools exist for detecting HRD based on mutational patterns. In this study, using sequencing data from 1,043 breast and 182 ovarian cancers, we trained Homologous Recombination Proficiency Profiler (HRProfiler), a machine learning method for detecting HRD using six mutational features. The performance of HRProfiler was assessed against prior approaches using additional independent datasets of 417 breast and 115 ovarian cancers, including retrospective data from a clinical trial involving patients treated with PARP inhibitors. Individual HRD-associated mutational signatures alone did not consistently detect HRD or predict clinical response across datasets. Notably, while all tools performed comparably for whole-genome–sequenced cancers, HRProfiler was the only approach that consistently identified HRD in whole-exome–sequenced breast and ovarian cancers, offering clinically relevant insights. Retrospective analyses provided strong evidence that HRProfiler could serve as a valuable tool for predicting HRD and clinical response in breast and ovarian cancers. This study provides the rationale for large-scale prospective clinical trials to validate the potential of HRProfiler as a routine predictive and/or prognostic HRD biomarker to guide clinical decision-making.

**Significance:**

HRProfiler is a machine learning approach that reliably identifies homologous recombination deficiency in whole-exome–sequenced breast and ovarian cancers, outperforming other tools and providing clinically useful insights.

This article is part of a special series: Driving Cancer Discoveries with Computational Research, Data Science, and Machine Learning/AI.

*See related commentary by Lim and Ju, p. 2348*

## Graphical Abstract

**Figure:**
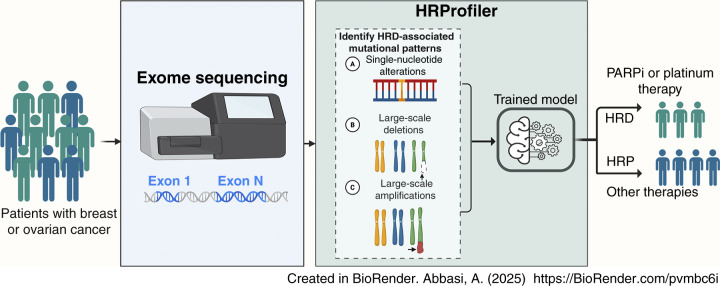

## Introduction

Repair of DNA double-strand breaks by homologous recombination is an essential cellular mechanism for maintaining genomic stability and preventing tumorigenesis (1). Prior studies have elucidated key genes in the homologous recombination pathway, including *BRCA1*, *BRCA2*, *RAD51*, and *PALB2*, that commonly have pathogenic germline variants and/or somatic mutations in breast and ovarian cancers (1). Defects in homologous recombination genes can disable the homologous recombination repair pathway, making cells vulnerable to double-strand breaks and thus providing a treatment opportunity. Specifically, patients with cancers harboring defective homologous recombination repair are sensitive to both PARP inhibitors (PARPi) and platinum chemotherapy (2, 3).

Conventional stratification of homologous recombination–deficient (HRD) and homologous recombination–proficient (HRP) cancers involves screening for canonical genomic markers, including pathogenic germline variants and somatic copy-number (CN) alterations in homologous recombination genes (4–6). Previous experimental studies (7) and genomic analyses (8) have also revealed that HRD cells exhibit characteristic patterns of somatic mutations because of the activities of HRD-associated mutational processes. Currently, there are at least seven mutational signatures that have been putatively associated with and/or utilized to detect HRD: (i) single-base substitution (SBS) signatures SBS3 and SBS8, which are generally flat signatures that have a nearly uniform distribution across the trinucleotide mutational channels yet maintain distinct profiles (9); (ii) genomic rearrangement signatures RS3 and RS5, reflecting nonclustered tandem duplications and deletions, respectively (10); (iii) small insertion and deletion (ID) signatures ID6 and ID8, predominately encompassing IDs at microhomologies (11); and (iv) CN signature CN17, characterized by large tandem duplications (12).

At least three machine learning approaches have also been developed to capture HRD cancers by examining the patterns of somatic mutations found in cancer genomes: HRDetect (13), CHORD (14), and SigMA (15). HRDetect uses signatures SBS3, SBS8, RS3, RS5, and IDs at microhomologies corresponding to ID6 and ID8 to detect HRD in breast cancers (13). CHORD is an alternative pan-cancer HRD prediction tool that does not rely on mutational signatures, but it rather uses 29 mutational features directly observed in cancer genomes (14). CHORD is more computationally efficient, and prior studies have shown that it has an almost identical performance to that of HRDetect (13). However, both CHORD and HRDetect use HRD-specific patterns of genomic rearrangements that can only be reliably detected from whole-genome sequencing (WGS) data (13, 14). By excluding genomic rearrangements, HRDetect can also be applied to whole-exome sequencing (WES) data, albeit, with significantly diminished performance (13). Conversely, CHORD’s implementation does not allow for the utilization of the WES cancer datasets. In contrast to CHORD and HRDetect, SigMA was developed to exclusively detect the HRD-associated signature SBS3 from WGS, WES, and targeted gene panel sequencing data, with SigMA’s focus being on panel sequencing data (15). Nevertheless, to be applied to a sample, SigMA requires at least five somatic mutations within the examined cancer (15). Based on Memorial Sloan Kettering Cancer Center’s Integrated Mutation Profiling of Actionable Cancer Targets (MSK-IMPACT) data (16), this limits SigMA’s applicability to approximately 37% of breast and ovarian cancers profiled with MSK-IMPACT–targeted gene panel.

In this article, we perform retrospective analyses to evaluate the clinical utility of canonical gene-based biomarkers, HRD-associated mutational signatures, and machine learning approaches to detect treatment-sensitive breast and ovarian cancers. Although the presence of an individual HRD-associated mutational signature is generally ineffective in detecting clinical response, existing machine learning tools can capture treatment sensitivity in WGS cancer datasets but, in most cases, are less effective in WES cancer datasets. To address this limitation, we developed the Homologous Recombination Proficiency Profiler (HRProfiler), a machine learning method that harnesses only six mutational features for detecting clinically useful HRD from both whole-genome– and whole-exome–sequenced breast and ovarian cancers. Our findings offer a pragmatic approach to detecting HRD in WES cancer datasets and underscore the importance of exercising caution when considering individual HRD-associated mutational signatures as clinical biomarkers.

## Materials and Methods

### Data sources and preprocessing

In this study, previously published datasets were used for all feature engineering, model development, and validation for both breast and ovarian cancers profiled using WGS and/or WES. For WGS, the mutation and CN calls for the 560 breast cancer dataset were obtained from ftp://ftp.sanger.ac.uk/pub/cancer/Nik-ZainalEtAl-560BreastGenomes/. The Sweden Cancerome Analysis Network—Breast clinical trial triple-negative breast cancer (TNBC) dataset, comprising 237 TNBC samples (17), was sourced from https://data.mendeley.com/datasets/2mn4ctdpxp/. Additionally, Pan-Cancer Analysis of Whole Genomes (PCAWG) breast cancer mutation and CN data were retrieved from the International Cancer Genome Consortium portal: https://dcc.icgc.org/releases/PCAWG, and metastatic breast cancer mutation and CN data were retrieved from the Hartwig Medical Foundation (18) to assess the effect of treatment on HRProfiler’s features.

For WES, The Cancer Genome Atlas (TCGA) breast and ovarian cancer sequencing data and somatic mutation calls were downloaded from the Genomic Data Commons portal (https://portal.gdc.cancer.gov/). Exome-specific CN calls were derived in-house using ASCAT: https://github.com/VanLoo-lab/ascat. Additionally, the MSK-IMPACT dataset (109 breast and 50 ovarian whole-exome–sequenced cancers) was obtained from the Database of Genotypes and Phenotypes (accession number: phs001783.v1.p1) and processed using an EnsembleVariantCallingPipeline: https://github.com/AlexandrovLab/EnsembleVariantCallingPipeline. Additionally, 25 PARPi-treated high-grade ovarian cancers were obtained from the Database of Genotypes and Phenotypes (accession number: phs003019) and processed using the EnsembleVariantCallingPipeline.

### Feature engineering for predicting HRD

As previously performed (5, 13, 14), a sample with an HRD score of at least 42 for breast cancer (5) and 63 for ovarian cancer (19) or one harboring a germline/somatic alteration in *BRCA1* or *BRCA2* was annotated as HRD for all training purposes. All other samples were annotated as HRP. To identify significantly enriched features in HRD and HRP samples, we generated the average mutational profiles based on proportions across the 96 SBS, 83 ID, and 48 CN mutational contexts. To determine the differences in channels at every resolution, we performed Fisher exact tests to evaluate if there is any statistically significant difference in the average proportion of a given channel between HRD and HRP samples. Significant channels were identified for all types of mutational contexts if their log_2_-fold change was greater than 0.75 for whole-genome samples and 0.25 for whole-exome samples, and their −log_10_(FDR adjusted *P* value) was greater than 3. Similar workflows were adopted for both whole-genome– and whole-exome–sequenced samples, and only channels significantly enriched across both were considered for the feature engineering process. At the single-base resolution, A[C>T]G, C[C>T]G, G[C>T]G, and T[C>T]G channels were consistently enriched across HRP samples in both whole-genome and whole-exome datasets. Due to the overlapping mutational context, these four channels were combined into a single feature termed N[C>T]G, where N represents any of the four nucleotide bases (A, C, T, or G). Similarly, A[C>G]T, C[C>G]T, G[C>G]T, and T[C>G]T were channels consistently enriched in HRD samples and were combined into a single feature N[C>G]T. At the ID resolution, 5:Del:M:1, 5:Del:M:2, 5:Del:M:3, 5:Del:M:4, and 5:Del:M:5 were significantly enriched channels in HRD samples that represent varying microhomology sequence lengths at the deletion sites of ≥5 bps. These ID channels were combined into a single feature: DEL.5.MH, where DEL.5 presents deletions ≥5 bp and MH indicates microhomology sequences. At the CN resolution, significant LOH events with segment sizes between 1 and 40 Mb were combined into a single feature: LOH:1–40Mb. Similarly, diploid or genome-doubled segments greater than 40 Mb, with a total copy number (TCN) state between 2 and 4, were combined into 2–4:HET:>40 Mb. Lastly, significant amplification events, characterized by a TCN state of at least 3 and segment sizes between 10 and 40 Mb, were grouped into a single feature: 3–9:HET:10–40 Mb.

### Training and comparing HRD detection methods in WGS cancer data

To train a model for predicting HRD at WGS resolution, we used samples from the 560 breast dataset. Only 371 of the 560 samples that were labeled as evaluated in the HRDetect publication (13) were considered. The majority of the 371 samples were treatment-naïve with 303 collected prior to treatment, 13 collected after treatment, and 55 lacking treatment information. The six features derived from the feature engineering step were extracted from the 371 samples and normalized using StandardScaler in Python’s sklearn package. The training was based on 371 breast samples, comprising 131 HRD and 240 HRP samples, and used a linear kernel support vector machine with L2 regularization. Next, 10-fold cross-validation was conducted to tune hyperparameters and obtain feature weights from the model. To test the model’s performance, we predicted HRD probabilities for 71 treatment-naïve TCGA breast samples that were sequenced at both whole-genome and whole-exome resolutions. Samples with an HRD probability of at least 0.50 were considered HRD. To validate the model on an external dataset, we predicted HRD probabilities for 237 TNBC samples and evaluated its performance against the ground truth. Among these samples, 149 received adjuvant chemotherapy, 38 were excluded, and 50 remained untreated. The performance of the model was assessed using machine learning metrics such as sensitivity, precision, and F_1_ score. To compare the performance of HRProfiler with other tools, the HRD annotations were determined for the 237 TNBC samples using HRDetect, CHORD, and SigMA.

### Training and comparing HRD detection methods in WES cancer data

To train a breast cancer–specific model for predicting HRD at whole-exome resolution, we used treatment-naïve samples from the TCGA breast cancer dataset. Only 743 samples that had HRD annotations were used for both training and testing. The six features derived from the feature engineering step were extracted as proportions, except for DEL.5.MH, which was extracted as absolute counts. All features were normalized using StandardScaler in Python’s sklearn package. The training was based on 672 breast samples that included 156 HRD and 516 HRP samples. Next, 10-fold cross-validation was conducted to tune hyperparameters and obtain feature weights from the model. The model’s performance was tested on the held-out 71 breast samples that were previously sequenced at both whole-genome and whole-exome resolution. Following HRD threshold optimization using the training data, samples with an HRD probability of 0.50 or higher were classified as HRD. To validate the model on an external dataset, we predicted HRD probabilities for 109 MSK-IMPACT breast cancer whole-exome–sequenced samples and evaluated the model’s performance using metrics such as sensitivity, precision, and F_1_ score against the ground truth. The WES model was also applied to the downsampled 237 TNBC samples. The whole-exome features for the 237 TNBC samples were derived by downsampling the ASCAT CN calls to segments that spanned the exonic regions. The mutation and ID calls were downsampled to whole-exome resolution using SigProfiler (20). To compare the performance of HRProfiler with other tools, HRD probabilities were also determined for SigMA and HRDetect.

To train an ovarian-specific model for predicting HRD at WES resolution, we used samples from the TCGA ovarian dataset. Only 222 samples that had HRD annotations were used for both training and testing. Analogous to training HRProfiler for whole-exome–sequenced breast cancers, the six features derived from the feature engineering step were extracted as proportions, except for DEL.5.MH, which was extracted as absolute counts. All features were normalized using StandardScaler in Python’s sklearn package. The training was based on 182 ovarian cancers that comprised 82 HRD and 100 HRP samples. Next, 10-fold cross-validation was conducted to tune hyperparameters and obtain feature weights from the model. The model’s performance was tested on the held-out 40 ovarian cancers that were sequenced at whole-exome resolution. Samples with an HRD probability of at least 0.50 were considered HRD. To validate the model on an external dataset, we predicted HRD probabilities for 50 MSK-IMPACT whole-exome–sequenced ovarian cancers and evaluated the model’s performance against the ground truth. To compare the performance of HRProfiler with other tools, HRD annotations were determined for the same samples by HRDetect and SigMA using the default breast WGS and ovarian WES pretrained models, respectively.

### Deriving HRD status based on HRD-associated signatures, genes, and tools

Germline and somatic mutations for *BRCA1* and *BRCA2* and, when available, gene expression and promoter methylation changes in *BRCA1* and *BRCA2* were incorporated for the *BRCA1/2* annotations. Specifically, for TCGA breast cancers, the *BRCA1/2* annotations were derived from Polak and colleagues (21). Conversely, for TCGA ovarian cancers, these annotations were derived from Steele and colleagues (12). For all other datasets, the *BRCA1/2* annotations were derived from their respective publications.

SigProfilerAssignment (version 0.1.2) was used to determine the presence of HRD-associated signatures SBS3, ID6, and CN17 (22) using the Catalogue Of Somatic Mutations In Cancer (version 3.4) reference signatures. A sample was classified as HRD-positive for a given HRD signature if it had at least one mutational event attributed to that signature.

HRDetect was performed using the signature.tools.lib (version 2.3.0) package in R, available at https://github.com/Nik-Zainal-Group/signature.tools.lib. The default HRD probability threshold of 0.70 was used for predicting the HRD status for whole-genome–sequenced samples. To execute HRDetect on the WES data, we utilized the pretrained WGS model for the prediction. The rearrangement signatures RS3 and RS5, which cannot be derived from the WES data, were set to zero, and the default probability threshold of 0.70 was applied for classifying whole-exome–sequenced cancers as HRD. We also trained exome-specific HRDetect models for breast and ovarian cancers using all HRDetect features except for the rearrangement signature features RS3 and RS5, which cannot be detected from the WES data. These models were trained on the same training datasets and ground truth used for HRProfiler. A linear support vector machine was used and optimized through cross-validation and hyperparameter tuning. Samples with an HRD probability threshold of 0.50 or higher, determined after threshold optimization on the training data, were classified as HRD in both breast and ovarian cancers.

CHORD was performed using the extractSigsChord function installed from GitHub: https://github.com/UMCUGenetics/CHORD/. It was executed using default settings, and a probability threshold of 0.50 was applied for classifying samples as HRD.

SigMA (version 2.0) was downloaded from GitHub: https://github.com/parklab/SigMA/archive/refs/tags/2.0.tar.gz, and it was performed using the run function for signature 3 (also known as SBS3) prediction. For WGS breast datasets, we used the following parameters when running SigMA: data = “wgs,” do_assign = T, do_mva = T, tumor_type = “breast,” and catalog_name = “cosmic_v3p2_inhouse,” and we utilized SigMA strict predictions (pass_mva_strict) for our analysis. When performing SigMA on the WES datasets, we followed the same procedure as for WGS datasets, except for the data and tumor-type parameters. For predicting the signature 3 status for TCGA datasets, the data parameter was set to “tcga_mc3”; otherwise, it was set to “seqcap” for all other WES and downsampled WES (dWES) datasets. The tumor-type parameter was set to “breast” for breast and “ovary” for ovarian WES data.

### Survival analysis

The survival analysis was conducted using the KaplanMeierFitter and CoxPHFitter function from the lifelines package in Python (23). Interval disease-free survival was analyzed to assess the outcomes in chemotherapy-treated patients from the 237 TNBC dataset. Progression-free survival was evaluated to examine the survival trends among 25 patients with high-grade ovarian cancer treated with PARPi. Additionally, disease-specific survival was used to evaluate the survival trends in patients with TCGA ovarian cancer undergoing platinum-based therapy. *P* values and HRs listed in the Kaplan–Meier plots are based on the *P* values derived from the Cox proportional hazards model adjusted by dichotomized age of diagnosis (below and above 50 years old) as well as tumor stage or grade.

### Statistics

All statistical analyses were conducted in Python using the scikit-learn package. All *P* values were corrected for multiple hypothesis testing using the Benjamini–Hochberg procedure, where applicable.

### Data availability

HRProfiler is an open-source tool, and it is freely available for academic use as a Python package at https://github.com/AlexandrovLab/HRProfiler. The pretrained models for breast and ovarian cancers, based on WGS and WES data, are provided as part of the tool. All other raw data are available upon request from the corresponding author.

## Results

### Feature engineering and model training of HRProfiler

**Figure 1.**
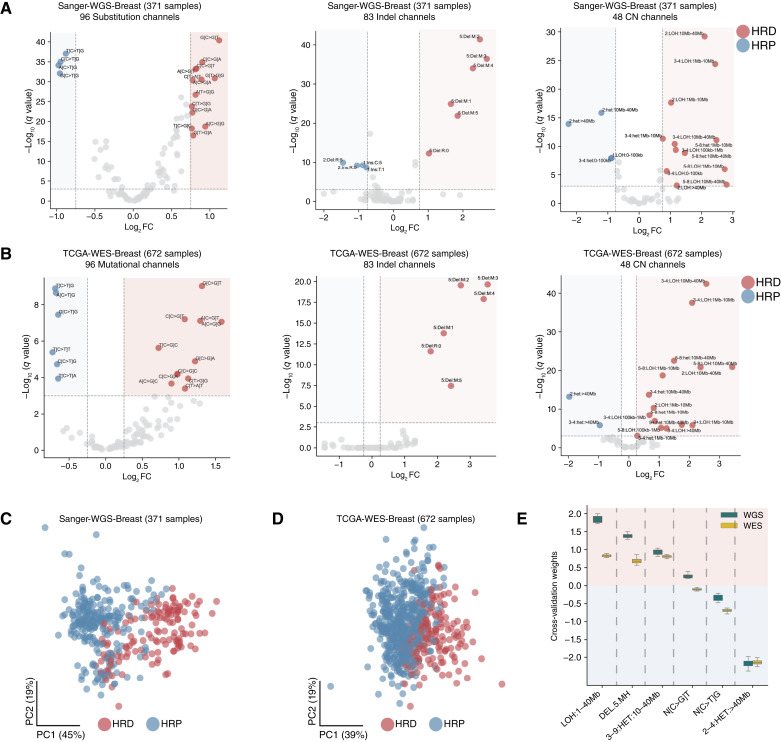
Feature engineering to identify significantly enriched somatic mutational features across HRD and HRP breast cancers. **A** and **B,** Volcano plots with log_2_-fold change (FC) enrichments across the average proportions of somatic mutations for 96 substitutions, 83 IDs, and 48 CN mutational channels between HRD and HRP cancers for 371 Sanger-WGS-Breast (**A**) and 672 TCGA-WES-Breast (**B**) samples. Channels with an absolute fold change greater than 0.75 for WGS data and 0.25 for WES data, and a −log_10_ FDR-adjusted *P* value >3 are colored. Channels colored in red are enriched in HRD samples, whereas channels highlighted in blue are enriched in HRP samples. **C** and **D,** Principal component (PC) analysis highlights the relevance of the features derived from the significant channels in **A** and **B** by separating HRD from HRP samples across the 371 Sanger-WGS-Breast (**C**) and 672 TCGA-WES-Breast (**D**) cohorts. **E,** The average 10-fold cross-validation weights of the six features derived from the WGS and WES breast training datasets using a linear kernel support vector machine. Positive weights reflect features predictive of HRD samples, whereas negative weights correspond to features predictive of HRP samples. Sanger-WGS-Breast, whole-genome–sequenced breast cancers using a subset of the Sanger Institute’s 560–breast cancer genome cohort; TCGA-WES-Breast, whole-exome–sequenced breast cancers using a subset of TCGA’s breast cancer cohort.

To determine a set of robust HRD-associated mutational patterns that can be detected using the WGS and WES cancer datasets, we identified significantly enriched mutation types specific to somatic SBSs (9), IDs (11), and CNs (12). In particular, using previously developed schemas (9, 11, 12), we compared the types of somatic mutations enriched in HRD or HRP cancers. Comparisons were performed for whole-genome–sequenced breast cancers using a subset of the Sanger Institute’s 560 breast cancer genome cohort (Fig. 1A; ref. 10) as well as for whole-exome–sequenced breast cancers using a subset of TCGA’s breast cancer cohort (Fig. 1B; ref. 24). Consistent with prior studies (13, 14, 25), a sample was classified as HRD for training purposes (i.e., ground truth) if it had an HRD score of at least 42 for breast cancer (5) or 63 for ovarian cancer (19) or if it harbored pathogenic germline variants, somatic mutations, or promoter methylation in *BRCA1* or *BRCA2*. Feature engineering and the subsequent training of HRProfiler were performed only on the designated training datasets (Supplementary Fig. S1).

At the SBS resolution, we observed a striking enrichment of C:G>T:A SBSs at the 5′-NpCpG-3′ context (mutated base is underlined; N reflects any base) in HRP samples (Fig. 1A and B). This suggests that a relatively large proportion of mutations in HRP samples are C:G>T:A transitions at CpG sites when compared with HRD samples. Conversely, HRD samples were enriched for C:G>G:C SBSs at the 5′-NpCpT-3′ context. At the ID resolution, we observed an enrichment of deletions spanning at least 5 bps with flanking microhomology sequences across HRD samples (Fig. 1A and B). These mutations are known to arise from the erroneous activities of the microhomology-mediated end joining or the single-strand annealing DNA repair pathways in the absence of a functional homologous recombination pathway (26). At the CN resolution, specific genomic alterations were enriched in HRD samples (Fig. 1A and B). LOH, a type of event in which one copy of a genomic region is lost, leaving only a single allele, was notably enriched in HRD samples for regions spanning 1 to 40 Mb. Additionally, heterozygous amplifications—events in which both alleles of a genomic region are retained but at least one is amplified—were observed to be enriched in HRD samples. These heterozygous amplifications spanned 10 to 40 Mb, with a TCN of both alleles ranging from 3 to 9. In contrast, very large (>40 Mb) heterozygous segments with a TCN between 2 and 4 were enriched in HRP samples (Fig. 1A and B). This finding suggests that very large diploid segments or regions that have undergone genome doubling are enriched in HRP samples, in line with the observation that HRP samples are genomically stable and harbor relatively low CN aberrations (25).

Based on these observations, we combined the mutational channels (as described in Materials and Methods) into six genomic features: (i) LOH:1–40 Mb; (ii) DEL.5.MH; (iii) 3–9:HET:10–40 Mb; (iv) C:G>G:C substitutions in the 5′-NpCpT-3′ context (N[C>G]T); (v) C:G>T:A substitutions in the 5′-NpCpG-3′ context (N[C>T]G); and (vi) 2–4:HET:>40 Mb. To evaluate if these genomic features are sufficient to distinguish HRD and HRP samples, we performed principal component analysis using the training data. We observed a separation between HRD and HRP samples across the two principal components for both WGS (Fig. 1C) and WES (Fig. 1D) breast cancer data.

To further evaluate the robustness of the six HRProfiler features in predicting HRD status beyond breast cancer, we analyzed two whole-exome–sequenced HRD-independent cancer types from TCGA—kidney renal clear-cell carcinoma and low-grade gliomas—as controls (27, 28). Principal component analysis revealed that as expected, kidney renal clear-cell carcinoma and low-grade glioma samples clustered almost exclusively with HRP breast cancers, whereas HRD and HRP breast cancer samples showed clear separation (Supplementary Fig. S2A and S2B). Additionally, to evaluate the effect of treatment on the six HRProfiler features, we analyzed the treatment status of WGS metastatic breast cancer data from the Hartwig Medical Foundation dataset (18) and observed no significant effect between treatment-naïve and treated cancers (Supplementary Fig. S3).

Next, using the six genomic features, we trained a machine learning tool, HRProfiler, based on a linear kernel support vector machine. HRProfiler comprises WGS and WES models that were trained using 371 samples from the dataset of whole-genome–sequenced breast cancers using a subset of the Sanger Institute’s 560 breast cancer genome cohort (13) and 672 samples from the dataset of whole-exome–sequenced breast cancers using a subset of TCGA’s breast cancer cohort (24), respectively (Supplementary Fig. S1). Ten-fold cross-validation was conducted to determine the feature weights for the two trained models. As expected, features with positive weights (i.e., LOH:1–40 Mb, DEL.5.MH, 3–9:HET:10–40 Mb, and N[C>G]T) were enriched in HRD samples, whereas features with negative weights (i.e., N[C>T]G and 2–4:HET:>40 Mb) were enriched in HRP samples (Fig. 1E).

### Comparing HRD detection methods in WGS and WES breast cancer data

In principle, two distinct approaches have been utilized to evaluate the performance of methods for detecting HRD. In their original publications, CHORD and HRDetect have relied on concordance between their predictions and prior HRD genomic annotations (13, 14). This concordance can be quantified by the area under the receiver operating characteristic curve (AUC) with both CHORD and HRDetect reporting AUCs above 0.90 for WGS cancer data (13, 14). However, this type of comparison requires a ground truth for HRD and HRP cancers, which, in most cases, is not straightforward to derive. The second approach relies on comparing clinical endpoints for HRD- and HRP-predicted cancers in patients treated with either chemotherapy or PARPi. The advantage of this approach is that it could provide immediate clinical relevance. Unfortunately, such comparisons require the availability of well-annotated clinicogenomic datasets, which are currently limited, especially at the whole-genome resolution. In this study, we utilized both approaches to put HRProfiler in the context of previously developed methods.

**Figure 2.**
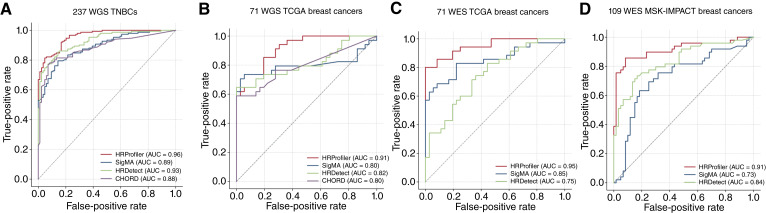
Performance of HRD tools on external validation datasets using HRD genomic ground truth annotations. ROC curves were derived for HRProfiler, SigMA, HRDetect, and CHORD. AUCs were calculated for each tool and are shown in the legends of the respective panels. **A,** ROCs for 237 whole-genome–sequenced TNBCs. **B,** ROCs for 71 whole-genome–sequenced TCGA breast cancers. **C,** ROCs for 71 whole-exome–sequenced breast cancers. **D,** ROCs for 109 whole-exome–sequenced MSK-IMPACT breast cancers. No ROCs are shown for CHORD in **C** and **D** as the tool cannot be applied to WES data. In all plots, the *x*-axes reflect the false-positive rates, whereas the *y*-axes correspond to the true-positive rates. Precision and recall curves for the same samples are provided in Supplementary Fig. S4.

To evaluate the performance of HRProfiler, SigMA, HRDetect, and CHORD in the context of HRD genomic ground truth annotations, we applied the four tools to an independent set of 237 whole-genome–sequenced TNBCs from the Sweden Cancerome Analysis Network—Breast project (ClinicalTrials.gov identifier NCT02306096; ref. 17), as well as to 71 held-out TCGA breast cancers, which have been profiled using both WGS and WES. Additionally, we applied the tools to an independent external WES dataset of 109 MSK-IMPACT breast cancers (29). All tools exhibited good AUC performance when applied to the WGS cancer data (Fig. 2A and B; Supplementary Fig. S4A and S4B), whereas HRProfiler outperformed HRDetect and SigMA for whole-exome–sequenced breast cancers (Fig. 2C and D; Supplementary Fig. S4C and S4D). CHORD could not be applied to WES data. Importantly, HRProfiler was the only tool with AUCs above 0.90 across all WES and WGS breast cancer datasets (Fig. 2).

**Figure 3.**
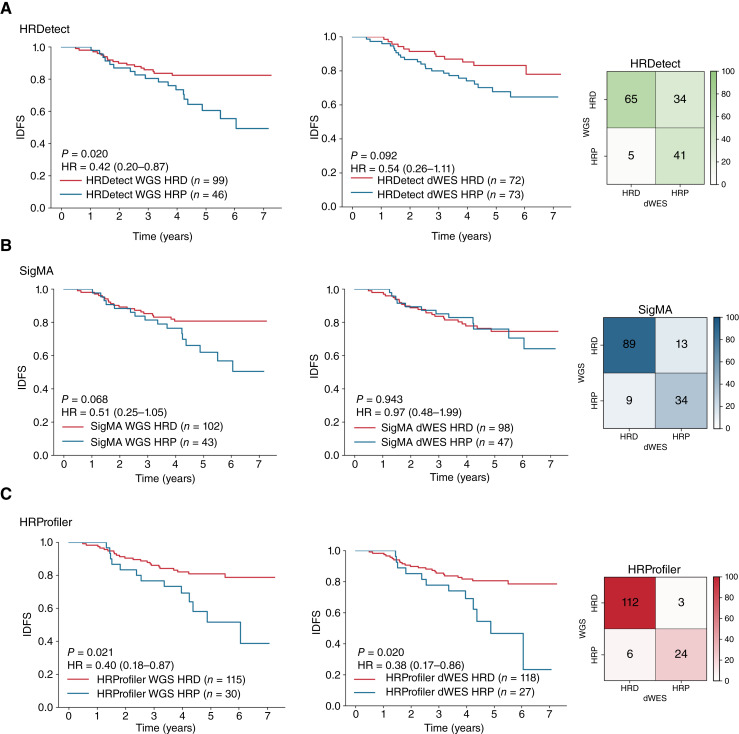
Predicting survival in breast cancers treated with chemotherapy by HRD tools. Kaplan–Meier curves and confusion matrices for samples predicted as HRD and HRP by HRDetect (**A**), SigMA (**B**), and HRProfiler (**C**) in 145 chemotherapy-treated TNBCs. In each panel, the left plot reflects the Kaplan–Meier curves for the WGS breast cancer dataset. The middle plot corresponds to the Kaplan–Meier curves for the same samples when downsampled to whole exomes. The right plot contains a confusion matrix that provides a comparison of each tool’s HRD annotations from WGS and dWES data. The *y*-axes on all Kaplan–Meier curves reflect interval disease-free survival (IDFS), and the *x*-axes correspond to time measured in years. Listed *P* values and HRs are based on a Cox proportional hazards model after adjusting for age at diagnosis and tumor grade. Within the Kaplan–Meier plots, 95% confidence intervals are provided for all HRs. The performance of CHORD on WGS data, which was almost identical to that of HRDetect, can be found in Supplementary Fig. S6. Comparisons of the clinical utility of BRCA1/2 defects and HRD-associated signatures SBS3, CN17, and ID6 for the same patients are provided in Supplementary Fig. S7.

To evaluate the potential clinical utility of HRProfiler, SigMA, HRDetect, and CHORD in serving as predictive biomarkers for adjuvant chemotherapy–treated breast cancers, we applied the tools to a subset of 145 whole-genome–sequenced chemotherapy-treated TNBCs with information for interval disease-free survival (17). Additionally, the 145 TNBCs were downsampled to whole exomes to further assess the ability of each tool to predict HRD robustly at both whole-genome and whole-exome resolutions. As previously reported (17), when applied to the WGS breast cancer dataset, HRDetect was able to identify 99 HRD samples, which exhibited better survival when compared with the 46 HRP samples after adjusting for grade and age at diagnosis (HR = 0.42; *P* value = 0.020; Fig. 3A). However, the tool exhibited markedly worse performance on the dWES data (HR = 0.54; *P* value = 0.092) with 39 samples (26.9% of all examined TNBCs) being differently annotated when compared with the WGS data. Using the original HRDetect model may not be optimal when examining WES data, as it was originally developed for WGS data and relies on rearrangement signatures. To address this, we trained a new WES breast cancer model using the HRDetect methodology, excluding rearrangement signatures and using the same ground truth as HRProfiler for a fair comparison. However, when applied to dWES breast cancer data, the new model did not exhibit improved performance (HR = 0.62; *P* value = 0.18; Supplementary Fig. S5A and S5B), suggesting that the rearrangement signatures may be crucial for HRDetect and/or highlighting challenges in detecting SBS3 and SBS8 from WES data (30).

CHORD’s performance on WGS cancer data was very similar to that of HRDetect (Supplementary Fig. S6A and S6B); however, the tool cannot be applied to the dWES data. Applying SigMA to the 145 TNBCs did not result in a statistically significant separation for either the WGS breast data (*P* value = 0.068) or the dWES data (*P* value = 0.94; Fig. 3B). In contrast, HRProfiler was able to better stratify breast cancers from both WGS (HR = 0.40; *P* value = 0.021) and dWES (HR = 0.38; *P* value = 0.02; Fig. 3C) data. Importantly, only nine samples (6.2% of all examined TNBCs) were differently annotated by HRProfiler when the tool was applied to WGS and dWES data (Fig. 3C). Lastly, partitioning the 145 TNBCs based on the presence of defects in *BRCA1/2* or the presence of HRD-associated signatures SBS3 or CN17 did not result in statistically significant separation (Supplementary Fig. S7). Nevertheless, stratifying the 145 TNBCs based on the presence of ID6 was able to separate the breast cancers albeit with diminished resolution (HR = 0.48; *P* value = 0.04; Supplementary Fig. S7).

To understand the discrepancies in HRD classification results between WGS and dWES data, we performed a targeted analysis of samples classified as HRD by HRProfiler, SigMA, and HRDetect using WGS data but as HRP by these tools when using dWES data, focusing on the differences in the features utilized by each tool for classification. For HRProfiler, a depletion of microhomology-mediated deletions in dWES data was observed in the three misclassified samples, likely explaining their HRP misclassification (Supplementary Fig. S8A). For SigMA, a reduced mutation count in dWES data impacted HRD detection, with 13 samples misclassified as HRP because of the low number of SBS3 mutations (Supplementary Fig. S8B). For HRDetect, the analysis of the 34 misclassified samples revealed differences in the activities of mutational signatures SBS3/e.3 and SBS8/e.8, as well as the absence of structural variations RS3 and RS5 in dWES data (Supplementary Fig. S8C).

### Comparing HRD detection methods in WES ovarian cancer data

To determine if the breast cancer–specific mutational features can be generalized to another HRD-associated cancer, we trained an ovarian-specific whole-exome model using 182 high-grade serous carcinomas from the TCGA-Ovarian-WES dataset (Supplementary Fig. S9A; ref. 24). As performed for breast cancer, 10-fold cross-validation was conducted for HRProfiler to determine the feature weights for the trained whole-exome model. Similar features to those observed in breast cancer were enriched in HRD and HRP ovarian cancers (Supplementary Fig. S9B). To examine the performance of HRProfiler, SigMA, and HRDetect in the context of HRD genomic ground truth annotations for whole-exome–sequenced ovarian cancer, we applied the three tools to 40 held-out TCGA ovarian samples as well as to an independent set of 50 MSK-IMPACT whole-exome–sequenced ovarian cancers (Supplementary Fig. S10A and S10B; ref. 29). For both datasets, HRProfiler outperformed the other two approaches by consistently exhibiting AUCs above 0.90 (Supplementary Fig. S10A and S10B).

**Figure 4.**
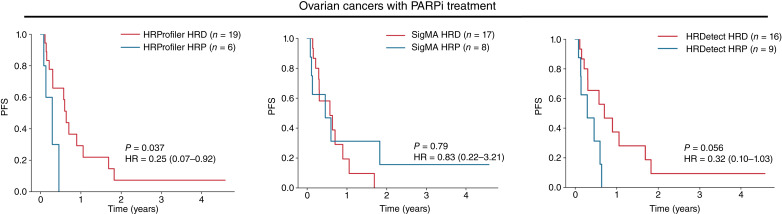
Predicting survival in ovarian cancers treated with PARPi by HRD tools. Kaplan–Meier curves for progression-free survival (PFS) across 25 PARPi-treated patients with high-grade serous ovarian cancer. Patients are annotated as HRD or HRP based on the predictions from HRProfiler (left), SigMA (middle), and HRDetect (right). Listed *P* values and HRs are based on a Cox proportional hazards model after adjusting for age at diagnosis and tumor stage. Within the Kaplan–Meier plots, 95% confidence intervals are provided for all HRs. Comparisons of the clinical utility of BRCA1/2 defects and HRD-associated signatures SBS3, CN17, and ID6 for the same patients are provided in Supplementary Fig. S12.

To assess the clinical utility of HRProfiler, SigMA, and HRDetect as predictors of clinical outcomes in ovarian cancer, we examined the progression-free survival for an independent set of 25 high-grade ovarian cancers from a phase Ib PARPi clinical trial of olaparib in combination with the PI3K inhibitor buparlisib (BKM120; ClinicalTrials.gov identifier NCT01623349; ref. 31). HRProfiler’s annotations were able to separate PARPi-treated samples based on progression-free survival (HR = 0.25; *P* value = 0.037; Fig. 4). The original WGS-trained HRDetect model also performed relatively well on these data (HR = 0.32; *P* value = 0.056; Fig. 4), whereas the exome-trained HRDetect ovarian cancer model exhibited worse performance (HR = 1.08; *P* value = 0.895; Supplementary Fig. S11A and S11B). It should be noted that annotating samples as HRD and HRP based on defects in *BRCA1/2* genes provided almost identical separation in progression-free survival for the 25 PARPi-treated ovarian cancers to that yielded by HRProfiler (Supplementary Fig. S12A). However, partitioning the 25 PARPi-treated ovarian cancers based on the presence of any of the HRD-associated signatures SBS3, CN17, or ID6 did not lead to differences in survival endpoints (Supplementary Fig. S12B and S12D).

Lastly, we extended our analysis to examine the platinum response in the held-out TCGA ovarian cohort of 32 platinum-treated samples, all profiled using WES (Supplementary Fig. S13A and S13B). In this cohort, HRProfiler was able to stratify patients based on clinical outcomes (HR = 0.23; *P* = 0.016), whereas SigMA, HRDetect, *BRCA1/2* mutation status, and HRD-associated mutational signatures showed no clinical separation (*P* values > 0.05).

## Discussion

There is increasing momentum in precision oncology toward more comprehensive genomic profiling to identify complex biomarkers such as HRD as part of routine clinical care (32). With continuing advances in sequencing technologies and the corresponding exponential decrease in their cost, clinical WES is becoming increasingly more prevalent (33–35). To harness the clinical utility of WES for predicting HRD, we present a novel machine learning approach called HRProfiler that utilizes a minimal set of six genomic features to predict HRD across both whole-genome– and whole-exome–sequenced breast and ovarian cancers. Unlike existing methods that focus solely on mutation types enriched in HRD samples (13–15), HRProfiler incorporates small- and large-scale mutational events enriched in both HRD and HRP cancers. HRProfiler also circumvents the need for genomic rearrangements and mutational signature extraction, which can be unreliable, especially when using sparse datasets derived from the WES data (30).

HRProfiler demonstrated comparable performance to existing approaches when applied to WGS data, and the tool surpassed other machine learning methods when applied to whole-exome–sequenced cancers. The suboptimal performance of HRDetect on whole-exome–sequenced tumors is perhaps unsurprising, given that HRDetect was developed for whole-genome–sequenced breast cancers, and the original publication noted poor performance for whole-exome–sequenced tumors (13). In contrast, despite its tailored design for WES and targeted panel sequencing data, SigMA exhibited comparatively limited performance in our tests. Indeed, SigMA is a machine learning surrogate for detecting HRD-associated signature SBS3, and our results show that SBS3 alone is not a reliable predictor of survival, even when detected by other tools. Similarly, other HRD-associated signatures, such as CN17 and ID6, did not provide consistent clinical separation for breast or ovarian cancers. Overall, these results indicate that the presence of an individual HRD-associated signature in a cancer sample does not necessarily indicate a clinically significant or actionable event.

We evaluated the HRProfiler’s performance using independent datasets, comprising 417 breast and 115 ovarian cancer samples, including data from two clinical trials. However, we acknowledge the limitations of using retrospective cohorts, particularly the potential for missing data or metadata, as well as the relatively small sample sizes for some of the reported survival analyses. In our HRD concordance analyses, we included all available samples with WES or WGS data, following previous methodologies (13–15). For the clinical endpoint analyses, only samples with complete clinical data for each patient were included. Specifically, for breast cancer, we conducted retrospective analyses on all treated samples with available WGS data that met the inclusion criteria of the NCT02306096 clinical trial (17). This clinical trial primarily focused on TNBC, which was utilized for validating our breast cancer model, given the higher prevalence of HRD in TNBC samples and the potential for patients in this subgroup to benefit from HRD-targeted therapies. For ovarian cancer, we focused on patients with ovarian cancer from the NCT01623349 clinical trial with available WES data (31). Future research should broaden this scope to include other breast cancer subtypes and other HRD-associated cancers, such as prostate and pancreatic cancers. Further validation through large-scale, well-designed prospective clinical trials will be essential to establish HRProfiler as a reliable predictive and prognostic biomarker for routine clinical decision-making. These studies should utilize extensive, balanced datasets of HRD-positive and HRD-negative samples and include comprehensive clinical annotations across various cancer types to ensure robust validation and broader applicability.

Building on these findings, it is important to consider how the composition of training data influences the performance of machine learning tools such as HRProfiler, particularly with respect to the class distribution of HRD and HRP samples. HRProfiler was trained on WGS breast cancer data containing 35% HRD samples, compared with HRDetect, which was trained on a dataset with 25% HRD samples. Despite these differences, both tools demonstrated nearly identical performance on WGS breast cancer data, suggesting that variations in class balance during training are likely not a key factor influencing their performance. For WES breast cancer data, HRProfiler, trained on data with 23% HRD samples, outperformed HRDetect, whose training data included 25% HRD samples but was not specifically optimized for WES. These results further confirm that the class distribution is unlikely to be a major factor for the observed performance differences.

Notwithstanding, HRProfiler provides a crucial link in utilizing the molecular phenotypic changes of impaired DNA repair mechanisms for detecting HRD in whole-exome–sequenced cancers. Moreover, the tool provides a robust and consistent approach that allows for detecting whole-exome–sequenced cancers that are sensitive to PARPi.

## Supplementary Material

Supplementary Figure S1 Supplementary Figure S1 illustrates the datasets used for training, testing, and validating HRProfiler in breast cancers.

Supplementary Figure S2 Supplementary Figure S2 demonstrates the robustness of HRProfiler features in discriminating HRD from HRP samples, independent of breast cancer type.

Supplementary Figure S3 Supplementary Figure S3 shows HRProfiler features are robust to the effects of treatment in breast cancers.

Supplementary Figure S4 Supplementary Figure S4 shows precision-recall curves comparing HRProfiler, SigMA, HRDetect, and CHORD across four breast cancer datasets using HRD genomic ground truth annotations.

Supplementary Figure S5 Supplementary Figure S5 displays survival curves comparing original and retrained HRDetect models on down-sampled breast cancers treated with chemotherapy.

Supplementary Figure S6 Supplementary Figure S6 presents CHORD-based survival predictions in chemotherapy-treated TNBC WGS samples.

Supplementary Figure S7 Supplementary Figure S7 evaluates the presence of defects in BRCA1/2 or HRD-associated signatures for predicting survival in chemotherapy-treated breast cancers.

Supplementary Figure S8 Supplementary Figure S8 highlights platform-specific differences in the features utilized by HRD tools for samples with discordant HRD classification.

Supplementary Figure S9 Supplementary Figure S9 illustrates the datasets used for training, testing, and validating HRProfiler in ovarian cancer.

Supplementary Figure S10 Supplementary Figure S10 displays performance of HRD tools on external ovarian validation datasets using HRD genomic ground truth annotations.

Supplementary Figure S11 Supplementary Figure S11 shows Kaplan-Meier survival curves comparing original and retrained HRDetect models in PARPi-treated ovarian cancers.

Supplementary Figure S12 Supplementary Figure S12 evaluates the presence of defects in BRCA1/2 or HRD-associated signatures for predicting survival in PARP inhibitor treated ovarian cancers.

Supplementary Figure S13 Supplementary Figure S13 evaluates the prediction of survival in platinum-treated TCGA ovarian samples across HRD tools, the presence of defects in BRCA1/2, and HRD-associated signatures.
