# Supplementary material for: HRProfiler Detects Homologous Recombination Deficiency in Breast and Ovarian Cancers Using Whole-Genome and Whole-Exome Sequencing Data
Source: Cancer Res. 2025 May 6;85(13):2504–13. doi: 10.1158/0008-5472.CAN-24-2639 (PMC12214882; doi:10.1158/0008-5472.CAN-24-2639)
Supplement: Supplementary Figure S1 — illustrates the datasets used for training, testing, and validating HRProfiler in breast cancers. [file can-24-2639_supplementary_figure_s1_suppsf1.pdf]

Supplementary Figure S1

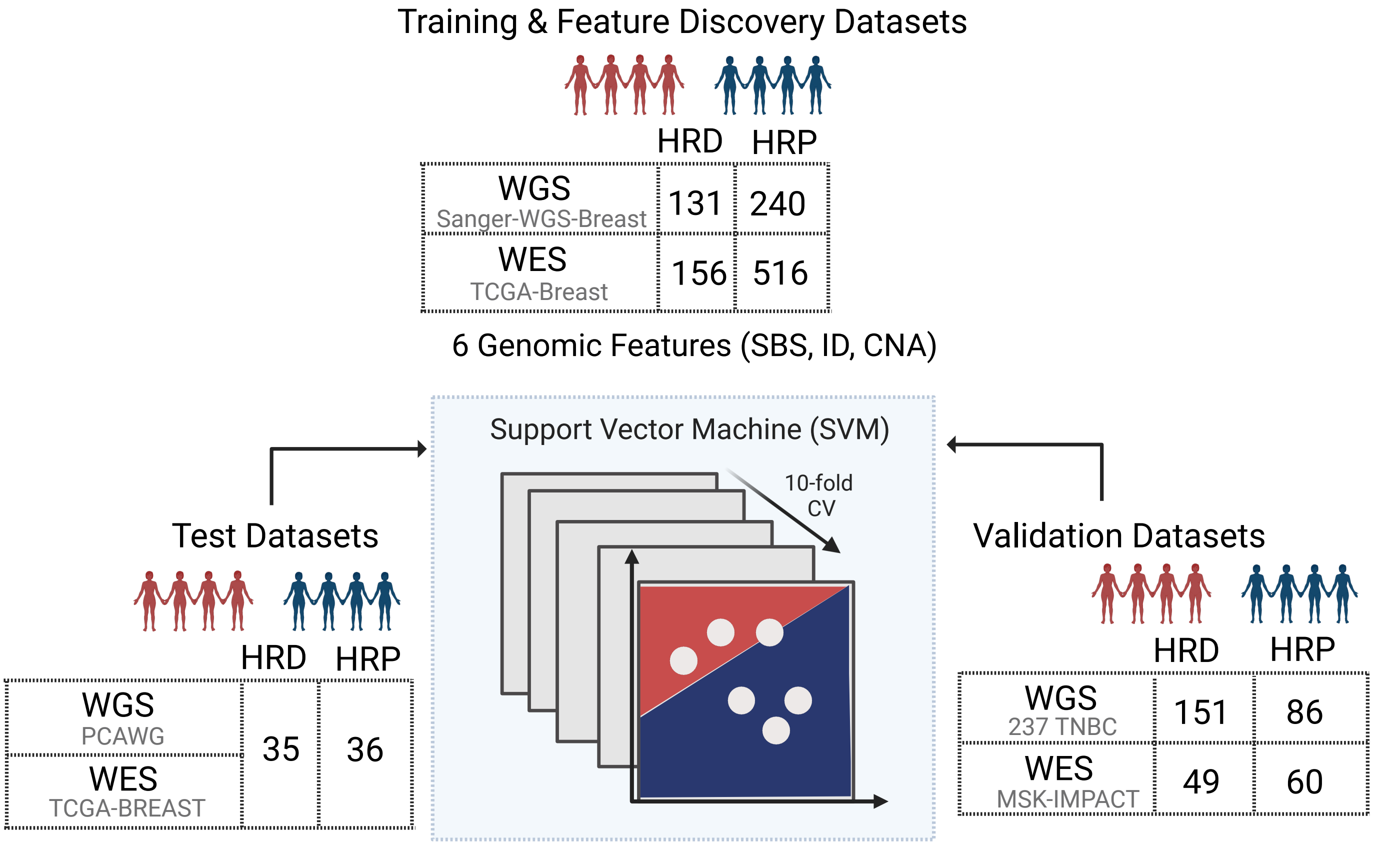

Supplementary Figure S1: Datasets used for training, testing, and validating HRProfiler in breast cancer. Schematic outline of the workflow for training, testing, and validating HRProfiler, a support vector machine model for detecting homologous recombination deficient (HRD) and homologous recombination proficient (HRP) breast cancers from whole-genome sequenced (WGS) and whole-exome sequenced (WES) data. The model was trained based on 6 genomic features, encompassing, single base substitutions (SBS), small insertions and deletions (ID), and copy-number alternations (CN). Training and testing data included samples from The Cancer Genome Atlas (TCGA), Sanger institute, and Pan-Cancer Analysis of Whole Genomes (PCAWG) study. Validation datasets include the independent Triple Negative Breast (TNBC) and the Memorial Sloan Kettering Cancer Center's Integrated Mutation Profiling of Actionable Cancer Targets (MSK-IMPACT) data.
