# Supplementary material for: HRProfiler Detects Homologous Recombination Deficiency in Breast and Ovarian Cancers Using Whole-Genome and Whole-Exome Sequencing Data
Source: Cancer Res. 2025 May 6;85(13):2504–13. doi: 10.1158/0008-5472.CAN-24-2639 (PMC12214882; doi:10.1158/0008-5472.CAN-24-2639)
Supplement: Supplementary Figure S2 — demonstrates the robustness of HRProfiler features in discriminating HRD from HRP samples, independent of breast cancer type. [file can-24-2639_supplementary_figure_s2_suppsf2.pdf]

Supplementary Figure S2

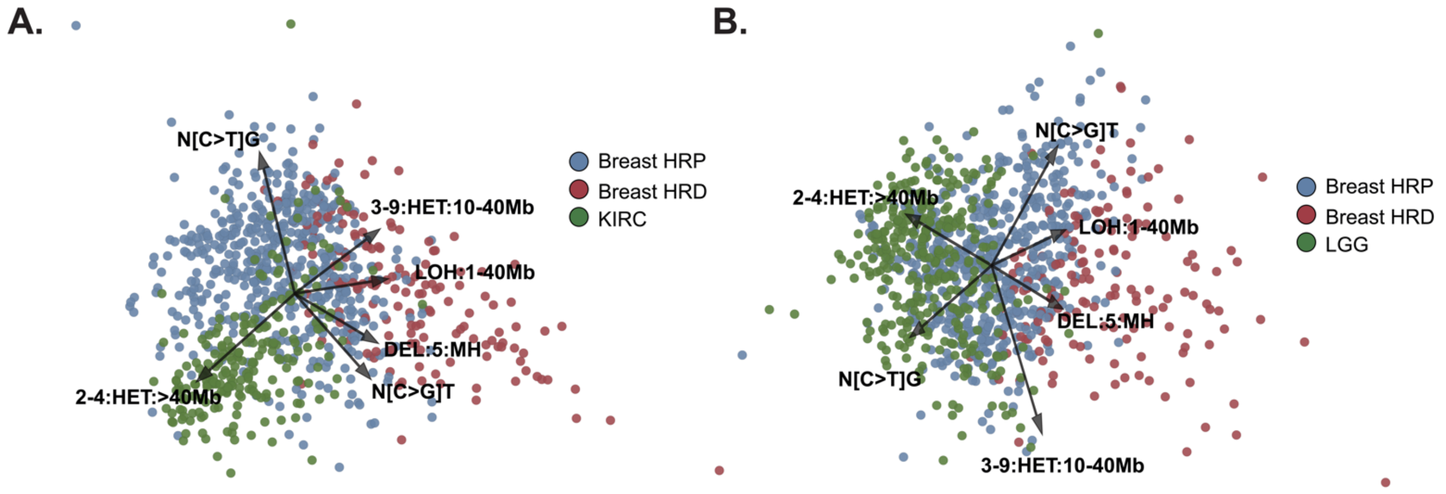

Supplementary Figure S2: Robustness of HRProfiler features in discriminating HRD from HRP samples, independent of breast cancer type. A principal component analysis biplot illustrating the six HRProfiler features across homologous recombination deficiency (HRD)-independent cancer types: ***(A)*** kidney renal clear cell carcinoma (KIRC) and ***(B)*** low-grade gliomas (LGG), in comparison with breast cancers classified as HRD or homologous recombination proficient (HRP). HRD and HRP breast cancer samples exhibit clear separation, while KIRC and LGG samples almost exclusively cluster with HRP breast cancers.
