# Supplementary material for: HRProfiler Detects Homologous Recombination Deficiency in Breast and Ovarian Cancers Using Whole-Genome and Whole-Exome Sequencing Data
Source: Cancer Res. 2025 May 6;85(13):2504–13. doi: 10.1158/0008-5472.CAN-24-2639 (PMC12214882; doi:10.1158/0008-5472.CAN-24-2639)
Supplement: Supplementary Figure S3 — shows HRProfiler features are robust to the effects of treatment in breast cancers. [file can-24-2639_supplementary_figure_s3_suppsf3.pdf]

Supplementary Figure S3

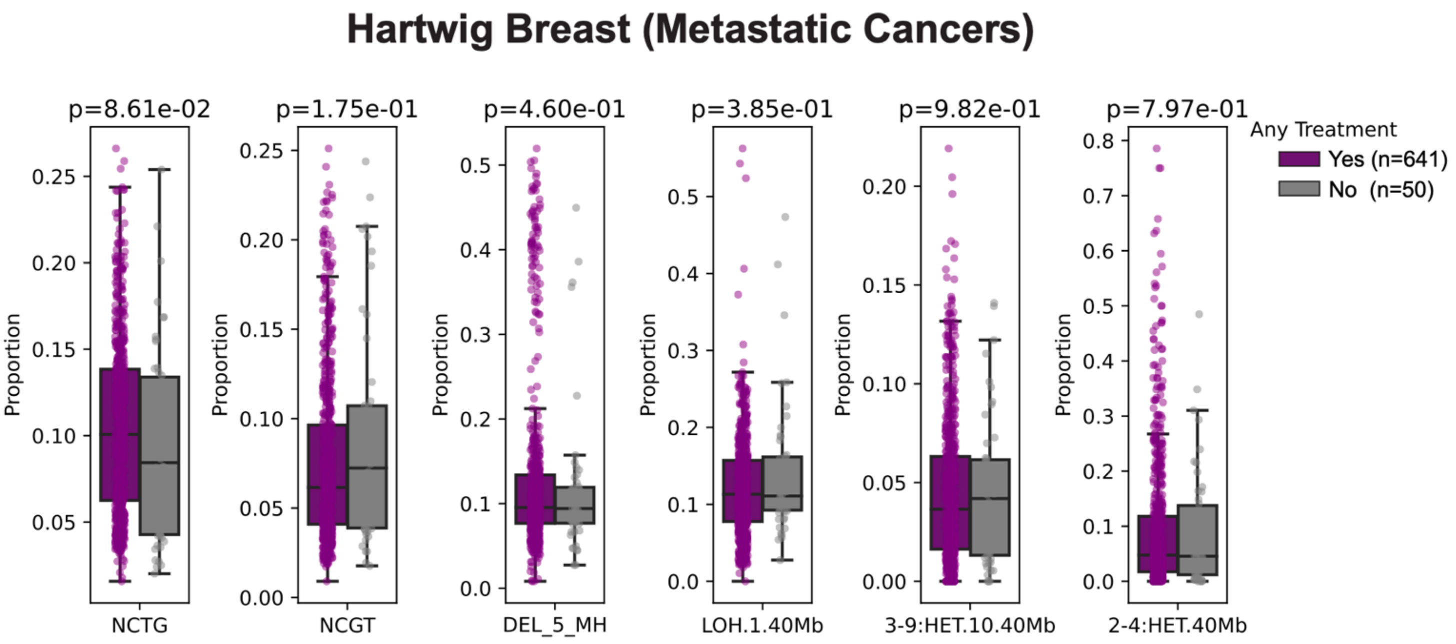

Supplementary Figure S3: HRProfiler features are robust to the effects of treatment in breast cancers. Distribution of the six HRProfiler features between treatment-naïve and treated WGS metastatic breast cancers from the Hartwig Medical Foundation (HMF) across all treatment modalities. Wilcoxon rank-sum test statistics were performed to determine differences between the two treatment groups and p-values are shown for each feature
