# Supplementary material for: HRProfiler Detects Homologous Recombination Deficiency in Breast and Ovarian Cancers Using Whole-Genome and Whole-Exome Sequencing Data
Source: Cancer Res. 2025 May 6;85(13):2504–13. doi: 10.1158/0008-5472.CAN-24-2639 (PMC12214882; doi:10.1158/0008-5472.CAN-24-2639)
Supplement: Supplementary Figure S4 — shows precision-recall curves comparing HRProfiler, SigMA, HRDetect, and CHORD across four breast cancer datasets using HRD genomic ground truth annotations. [file can-24-2639_supplementary_figure_s4_suppsf4.pdf]

# Supplementary Figure S4

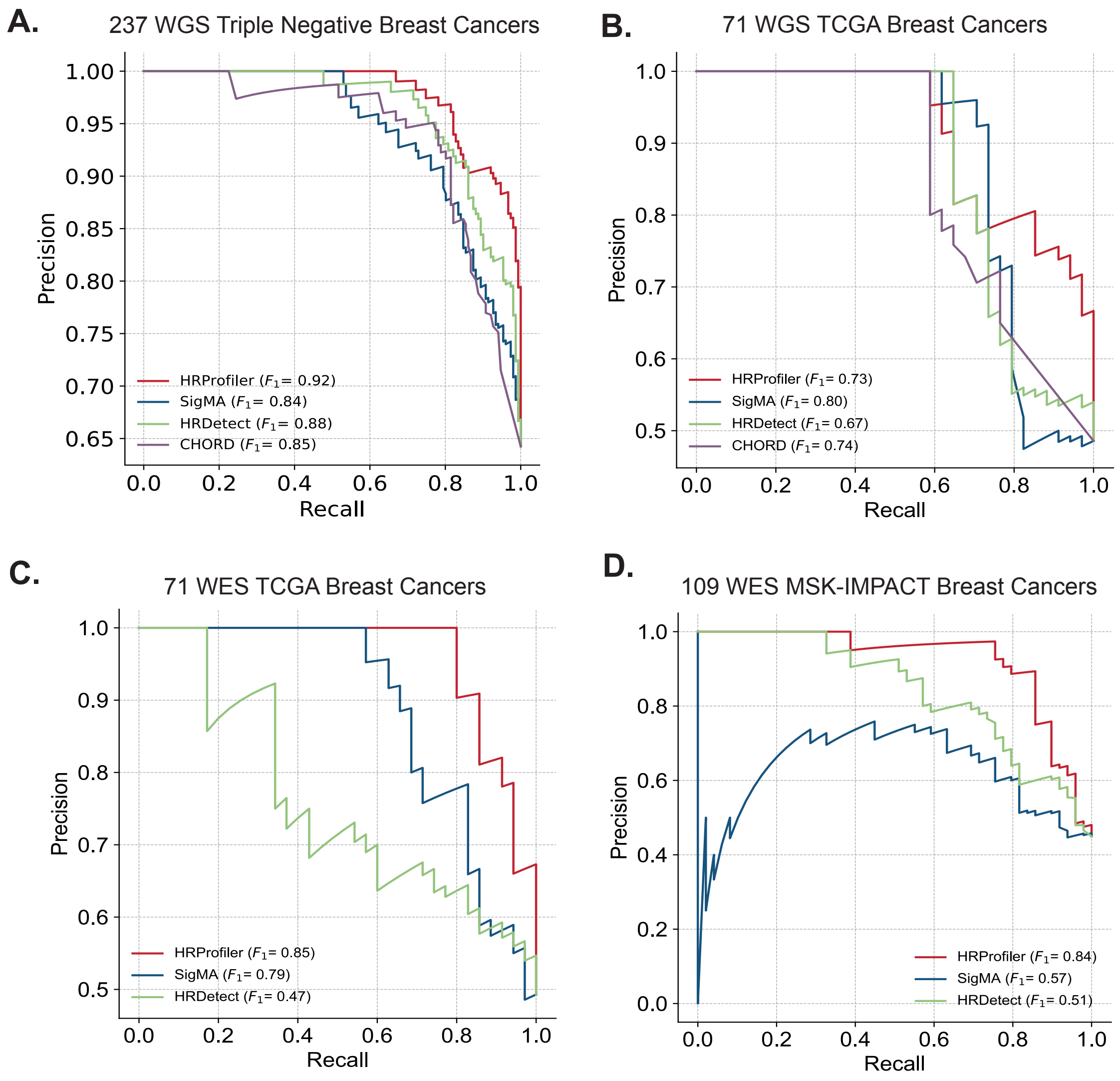


**Supplementary Figure S4: Precision and recall of HRD tools on breast validation datasets using HRD genomic ground truth annotations.** Precision and recall (PR) curves were calculated for HRProfiler, SigMA, HRDetect, and CHORD. ***(A)*** PR curves for 237 whole-genome sequenced (WGS) triple negative breast cancers. ***(B)*** PR curves for 71 WGS breast cancers from The Cancer Genome Atlas (TCGA) project. ***(C)*** PR curves for 71 whole-exome sequenced (WES) breast cancers. ***(D)*** PR curves for 109 MSK-IMPACT whole-exome sequenced breast cancers. No PR curves are shown for CHORD in panels *(C)* and *(D)* as the tool cannot be applied to WES data. The $F_1$ scores, *i.e.*, the harmonic mean of precision and recall, are shown for each tool within the respective panel's legend.
