# Supplementary material for: HRProfiler Detects Homologous Recombination Deficiency in Breast and Ovarian Cancers Using Whole-Genome and Whole-Exome Sequencing Data
Source: Cancer Res. 2025 May 6;85(13):2504–13. doi: 10.1158/0008-5472.CAN-24-2639 (PMC12214882; doi:10.1158/0008-5472.CAN-24-2639)
Supplement: Supplementary Figure S5 — displays survival curves comparing original and retrained HRDetect models on down-sampled breast cancers treated with chemotherapy. [file can-24-2639_supplementary_figure_s5_suppsf5.pdf]

Supplementary Figure S5

A.

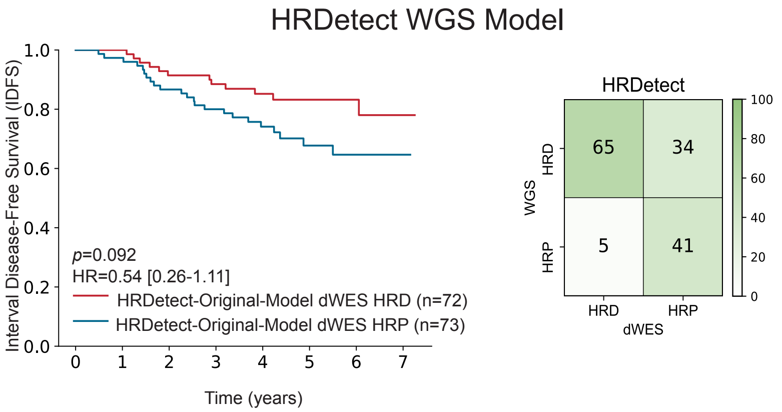

B.

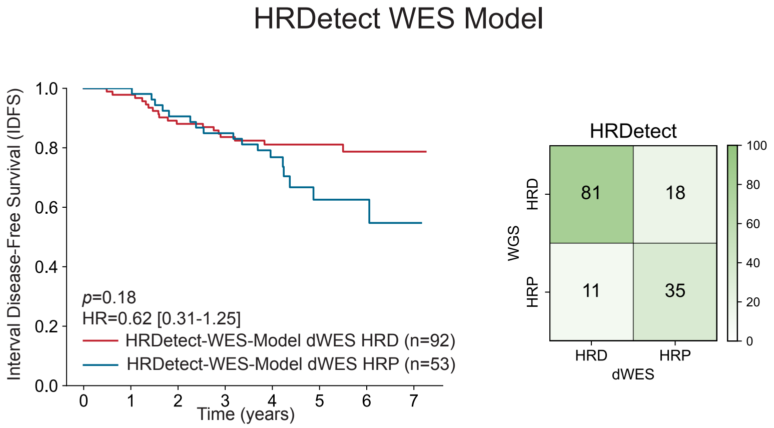

Supplementary Figure S5: Predicting survival in breast cancers treated with chemotherapy with different HRDetect models. ***(A)*** The performance of the original HRDetect model when applied to the down-sampled whole-exome sequencing (dWES) data. ***(B)*** The performance of the newly trained HRDetect whole-exome sequencing (WES) model when applied to dWES data. In each panel, the left plot reflects the Kaplan-Meier curves for dWES breast cancer data. The right plot corresponds to a confusion matrix that provides a comparison between each HRDetect model when applied to dWES data and the original HRDetect model when applied to whole-genome sequencing (WGS) breast cancer data. The y-axes on all Kaplan-Meier curves reflect Interval Disease Free Survival (IDFS), and the x-axes correspond to time measured in years. Listed p-values and hazard ratios (HRs) are based on a Cox proportional hazards model after adjusting for age at diagnosis and tumor grade. 95% confidence intervals are provided for all HRs within the Kaplan-Meier plots.
