# Supplementary material for: HRProfiler Detects Homologous Recombination Deficiency in Breast and Ovarian Cancers Using Whole-Genome and Whole-Exome Sequencing Data
Source: Cancer Res. 2025 May 6;85(13):2504–13. doi: 10.1158/0008-5472.CAN-24-2639 (PMC12214882; doi:10.1158/0008-5472.CAN-24-2639)
Supplement: Supplementary Figure S6 — presents CHORD-based survival predictions in chemotherapy-treated TNBC WGS samples. [file can-24-2639_supplementary_figure_s6_suppsf6.pdf]

Supplementary Figure S6

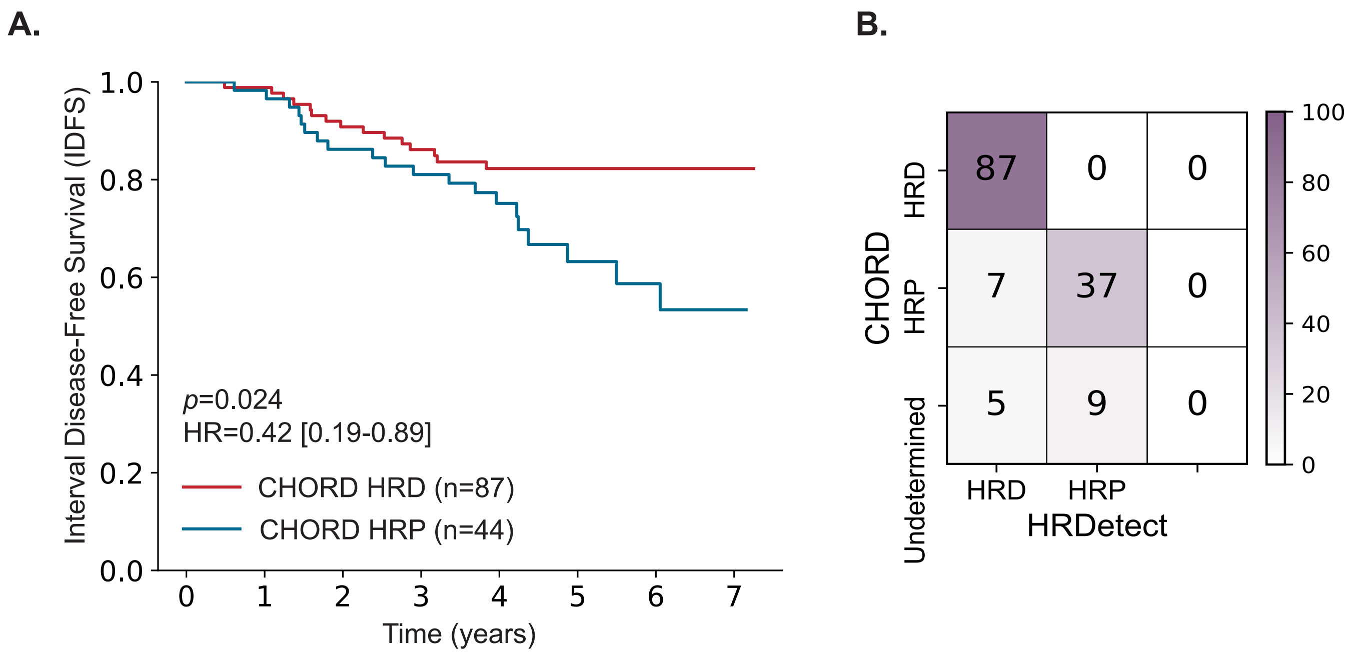

Supplementary Figure S6: Evaluating CHORD for predicting survival to chemotherapy in whole-genome sequenced breast cancers. All presented results are for 145 chemotherapy-treated whole-genome sequenced (WGS) triple negative breast cancers. ***(A)*** Kaplan-Meier curves for 131 breast cancers annotated as HRD and HRP by CHORD. Note that 14/145 samples were classified as undetermined by CHORD and these samples were excluded from the survival analysis. The y-axis of the Kaplan-Meier curves reflects Interval Disease Free Survival (IDFS), and the x-axis corresponds to time measured in years. The p-value and hazard ratio (HR) are based on a Cox proportional hazards model after adjusting for age and tumor grade. An 95% confidence interval is provided for the HR within the Kaplan-Meier plot. ***(B)*** Confusion matrix comparing the HRD and HRP annotations between CHORD and HRDetect.
