# Supplementary material for: HRProfiler Detects Homologous Recombination Deficiency in Breast and Ovarian Cancers Using Whole-Genome and Whole-Exome Sequencing Data
Source: Cancer Res. 2025 May 6;85(13):2504–13. doi: 10.1158/0008-5472.CAN-24-2639 (PMC12214882; doi:10.1158/0008-5472.CAN-24-2639)
Supplement: Supplementary Figure S7 — evaluates the presence of defects in BRCA1/2 or HRD-associated signatures for predicting survival in chemotherapy-treated breast cancers. [file can-24-2639_supplementary_figure_s7_suppsf7.pdf]

Supplementary Figure S7

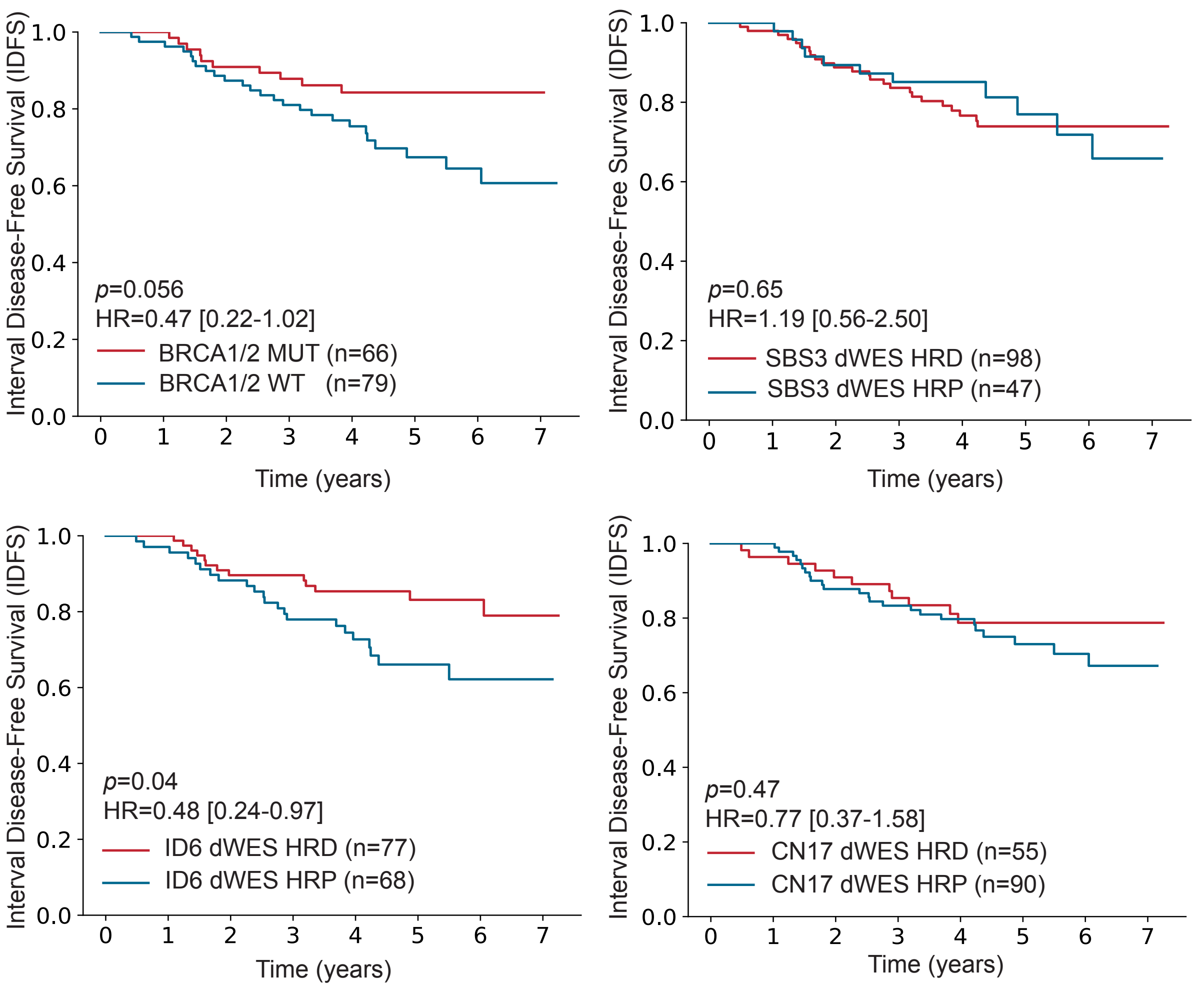

Supplementary Figure S7: Evaluating the presence of defects in *BRCA1/2* or HRD-associated signatures for predicting survival in chemotherapy-treated breast cancers. All presented results are for 145 chemotherapy-treated triple negative breast cancers down-sampled to whole-exomes (dWES). Kaplan-Meier curves and hazard ratios (HRs) for samples annotated as HRD and HRP by either a defect in *BRCA1/2* or by the presence of HRD-associated signatures SBS3, CN17, or ID6. The p-values and HRs are based on a Cox proportional hazards model after adjusting for age and tumor grade. 95% confidence intervals are provided for the HRs within the Kaplan-Meier plots. The y-axes on all Kaplan-Meier curves reflect Interval Disease Free Survival (IDFS), and the x-axes correspond to time measured in years.
