# Supplementary material for: HRProfiler Detects Homologous Recombination Deficiency in Breast and Ovarian Cancers Using Whole-Genome and Whole-Exome Sequencing Data
Source: Cancer Res. 2025 May 6;85(13):2504–13. doi: 10.1158/0008-5472.CAN-24-2639 (PMC12214882; doi:10.1158/0008-5472.CAN-24-2639)
Supplement: Supplementary Figure S8 — highlights platform-specific differences in the features utilized by HRD tools for samples with discordant HRD classification. [file can-24-2639_supplementary_figure_s8_suppsf8.pdf]

Supplementary Figure S8

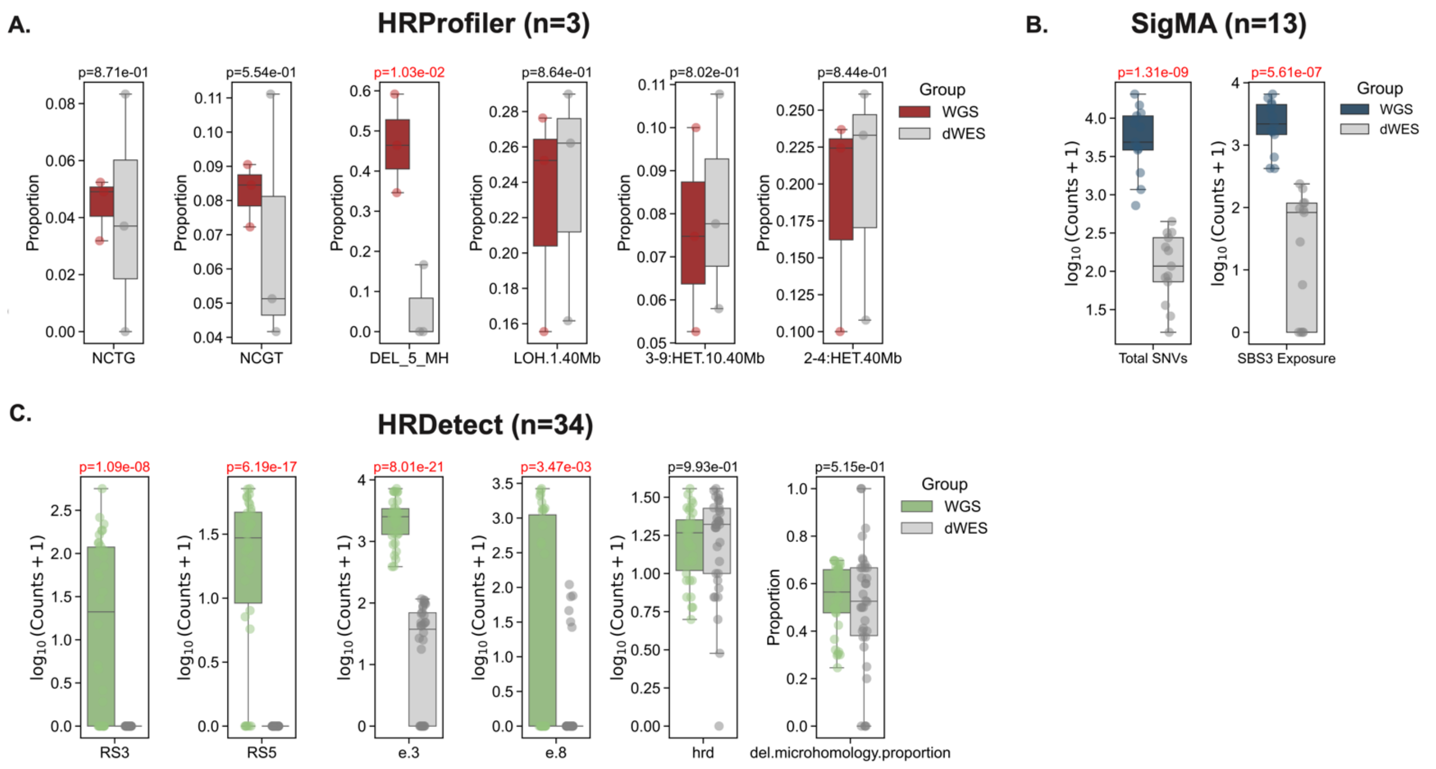

Supplementary Figure S8: Platform-specific differences in the features utilized by HRD tools for samples with discordant HRD classification. ***(A)*** HRProfiler: Box plots display the distribution of six HRProfiler input features for three samples classified as HRD in WGS but as HRP in dWES. ***(B)*** SigMA: Box plots show total SNV counts and SBS3 exposure, the primary features for SigMA, across 13 samples classified as HRD in WGS but as HRP in dWES. ***(C)*** HRDetect: Box plots present six HRDetect input features, including rearrangement signatures (RS3, RS5), SBS-related mutational signatures (SBS3/e.3, SBS8/e.8), Loss of Heterozygosity (LOH), and microhomology-mediated deletions, across 34 samples classified as HRD in WGS but HRP in dWES. Each box plot compares WGS (colored) and dWES (gray) data, with p-values indicating the statistical significance of differences between the platforms. Statistically significant p-values are highlighted in red.
