# Supplementary material for: HRProfiler Detects Homologous Recombination Deficiency in Breast and Ovarian Cancers Using Whole-Genome and Whole-Exome Sequencing Data
Source: Cancer Res. 2025 May 6;85(13):2504–13. doi: 10.1158/0008-5472.CAN-24-2639 (PMC12214882; doi:10.1158/0008-5472.CAN-24-2639)
Supplement: Supplementary Figure S9 — illustrates the datasets used for training, testing, and validating HRProfiler in ovarian cancer. [file can-24-2639_supplementary_figure_s9_suppsf9.pdf]

Supplementary Figure S9

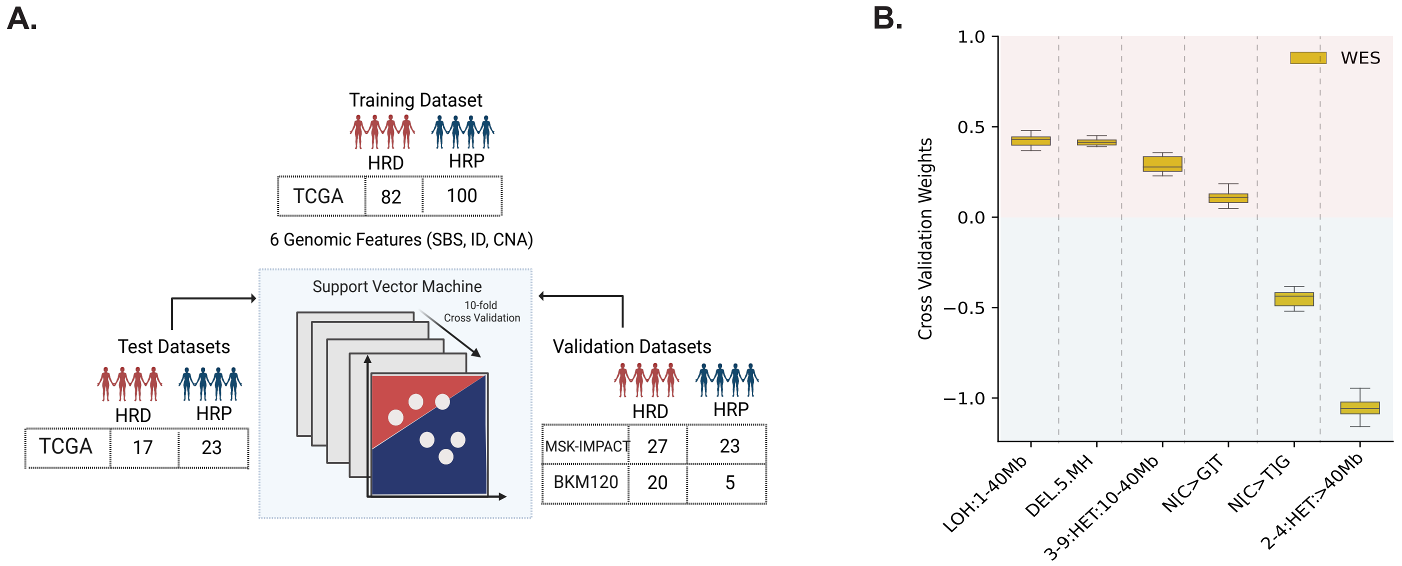

Supplementary Figure S9: Datasets and features used for training, testing, and validating HRProfiler in ovarian cancer. ***(A)*** Schematic outline of the workflow for training, testing, and validating HRProfiler, a support vector machine model for detecting homologous recombination deficient (HRD) and homologous recombination proficient (HRP) ovarian cancers from whole-exome sequenced data. The model was trained based on 6 genomic features, encompassing, single base substitutions (SBS), small insertions and deletions (ID), and copy-number alternations (CN). Training and testing data included samples from The Cancer Genome Atlas (TCGA) project. Validation datasets include the independent Memorial Sloan Kettering Cancer Center's Integrated Mutation Profiling of Actionable Cancer Targets (MSK-IMPACT) dataset and samples from a phase Ib trial of the PARP inhibitor olaparib in combination with the PI3K inhibitor (BKM120 cohort). ***(B)*** The average 10-fold cross validation weights of the six features derived from WES ovarian training dataset using a linear-kernel support vector machine. Positive weights reflect features predictive for HRD samples, while negative weights correspond to features predictive for HRP samples
