# Supplementary material for: HRProfiler Detects Homologous Recombination Deficiency in Breast and Ovarian Cancers Using Whole-Genome and Whole-Exome Sequencing Data
Source: Cancer Res. 2025 May 6;85(13):2504–13. doi: 10.1158/0008-5472.CAN-24-2639 (PMC12214882; doi:10.1158/0008-5472.CAN-24-2639)
Supplement: Supplementary Figure S10 — displays performance of HRD tools on external ovarian validation datasets using HRD genomic ground truth annotations. [file can-24-2639_supplementary_figure_s10_suppsf10.pdf]

Supplementary Figure S10

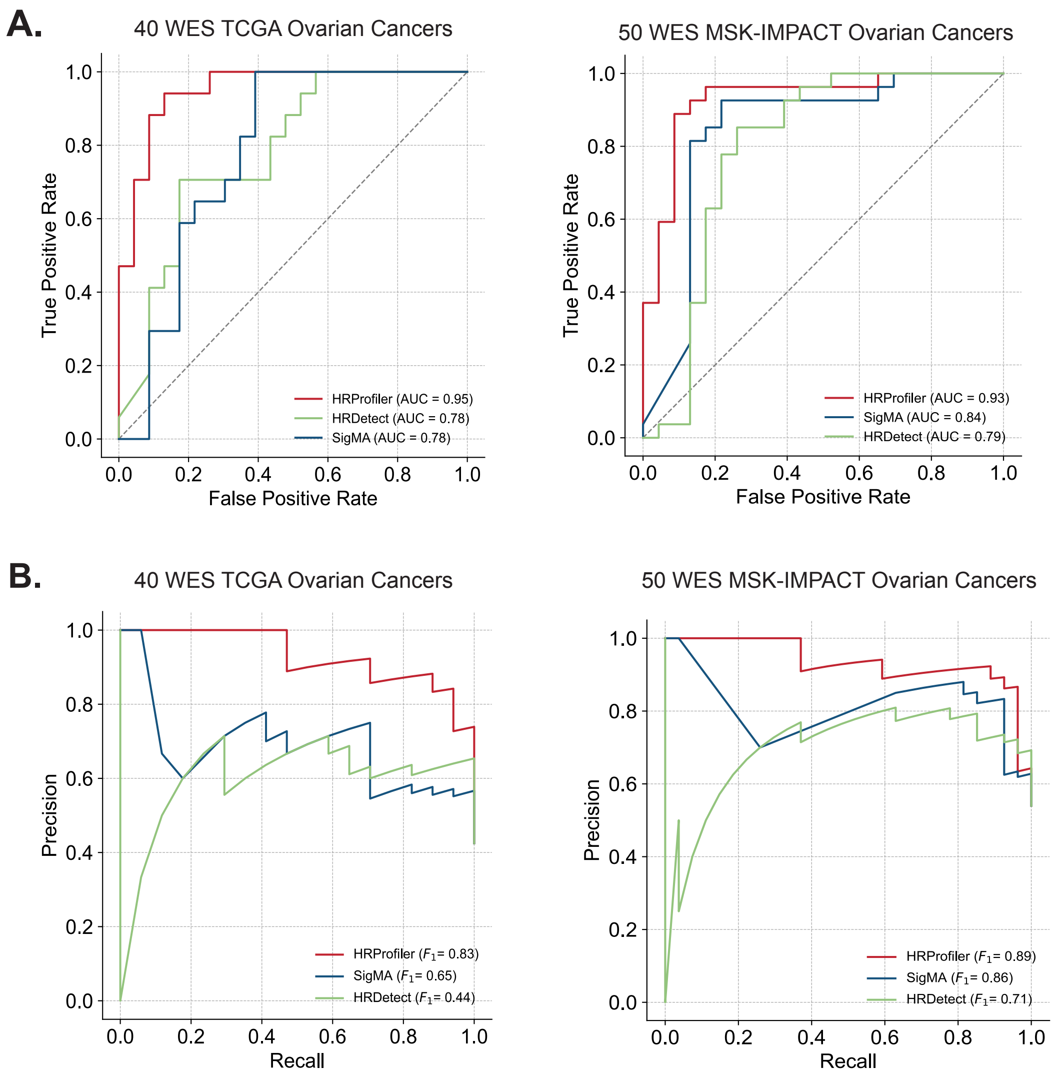

Supplementary Figure S10: Performance of HRD tools on external ovarian validation datasets using HRD genomic ground truth annotations. ***(A)*** Receiver operating characteristic (ROC) curves and ***(B)*** precision and recall curves were calculated for HRProfiler, SigMA, and HRDetect on a held-out test dataset of 40 whole-exome sequenced (WES) ovarian samples from The Cancer Genome Atlas (TCGA) project and on an external validation dataset of 50 whole-exome sequenced MSK-IMPACT ovarian cancers. The areas under the ROC (AUCs) as well as the F_1 scores, *i.e.*, the harmonic mean of precision and recall, are shown for each tool within the respective legend of each panel.
