# Supplementary material for: HRProfiler Detects Homologous Recombination Deficiency in Breast and Ovarian Cancers Using Whole-Genome and Whole-Exome Sequencing Data
Source: Cancer Res. 2025 May 6;85(13):2504–13. doi: 10.1158/0008-5472.CAN-24-2639 (PMC12214882; doi:10.1158/0008-5472.CAN-24-2639)
Supplement: Supplementary Figure S11 — shows Kaplan-Meier survival curves comparing original and retrained HRDetect models in PARPi-treated ovarian cancers. [file can-24-2639_supplementary_figure_s11_suppsf11.pdf]

Supplementary Figure S11

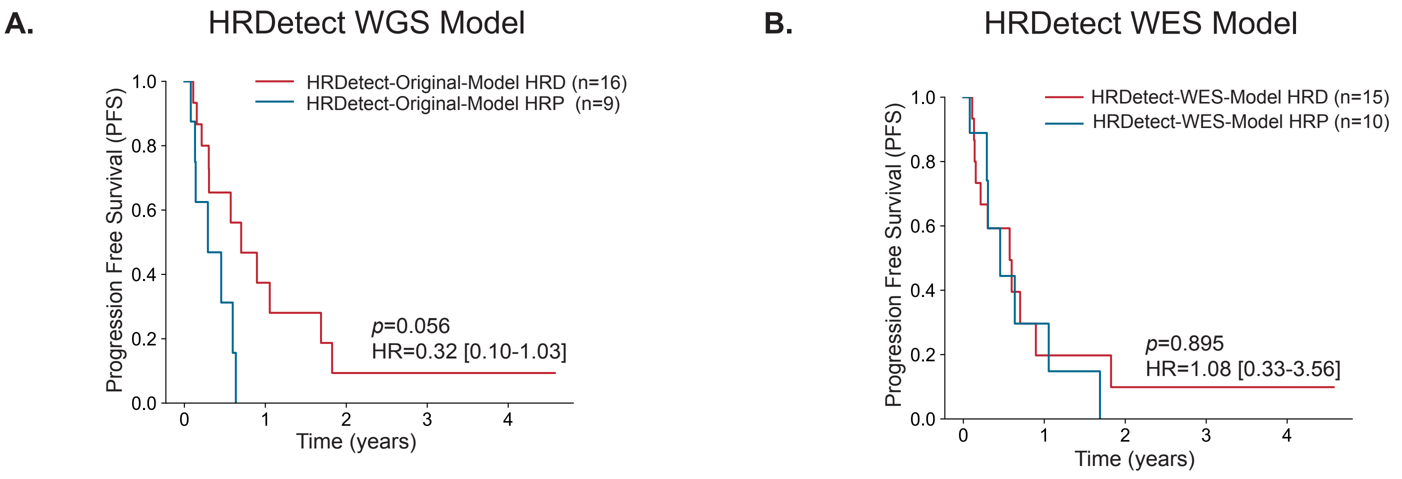

Supplementary Figure S11: Predicting survival in PARP inhibitor treated ovarian cancers with different HRDetect models. ***(A)*** The performance of the original HRDetect model when applied to the 25 whole-exome sequencing (WES) ovarian data. ***(B)*** The performance of the newly trained HRDetect WES model when applied to the same data. Each panel reflects the Kaplan-Meier curves for WES ovarian cancer data. The y-axes on all Kaplan-Meier curves reflect Progression Free Survival (PFS), and the x-axes correspond to time measured in years. Listed p-values and hazard ratios (HRs) are based on a Cox proportional hazards model after adjusting for age at diagnosis and tumor grade. 95% confidence intervals are provided for all HRs within the Kaplan-Meier plots**.**
