# Supplementary material for: HRProfiler Detects Homologous Recombination Deficiency in Breast and Ovarian Cancers Using Whole-Genome and Whole-Exome Sequencing Data
Source: Cancer Res. 2025 May 6;85(13):2504–13. doi: 10.1158/0008-5472.CAN-24-2639 (PMC12214882; doi:10.1158/0008-5472.CAN-24-2639)
Supplement: Supplementary Figure S12 — evaluates the presence of defects in BRCA1/2 or HRD-associated signatures for predicting survival in PARP inhibitor treated ovarian cancers. [file can-24-2639_supplementary_figure_s12_suppsf12.pdf]

Supplementary Figure S12

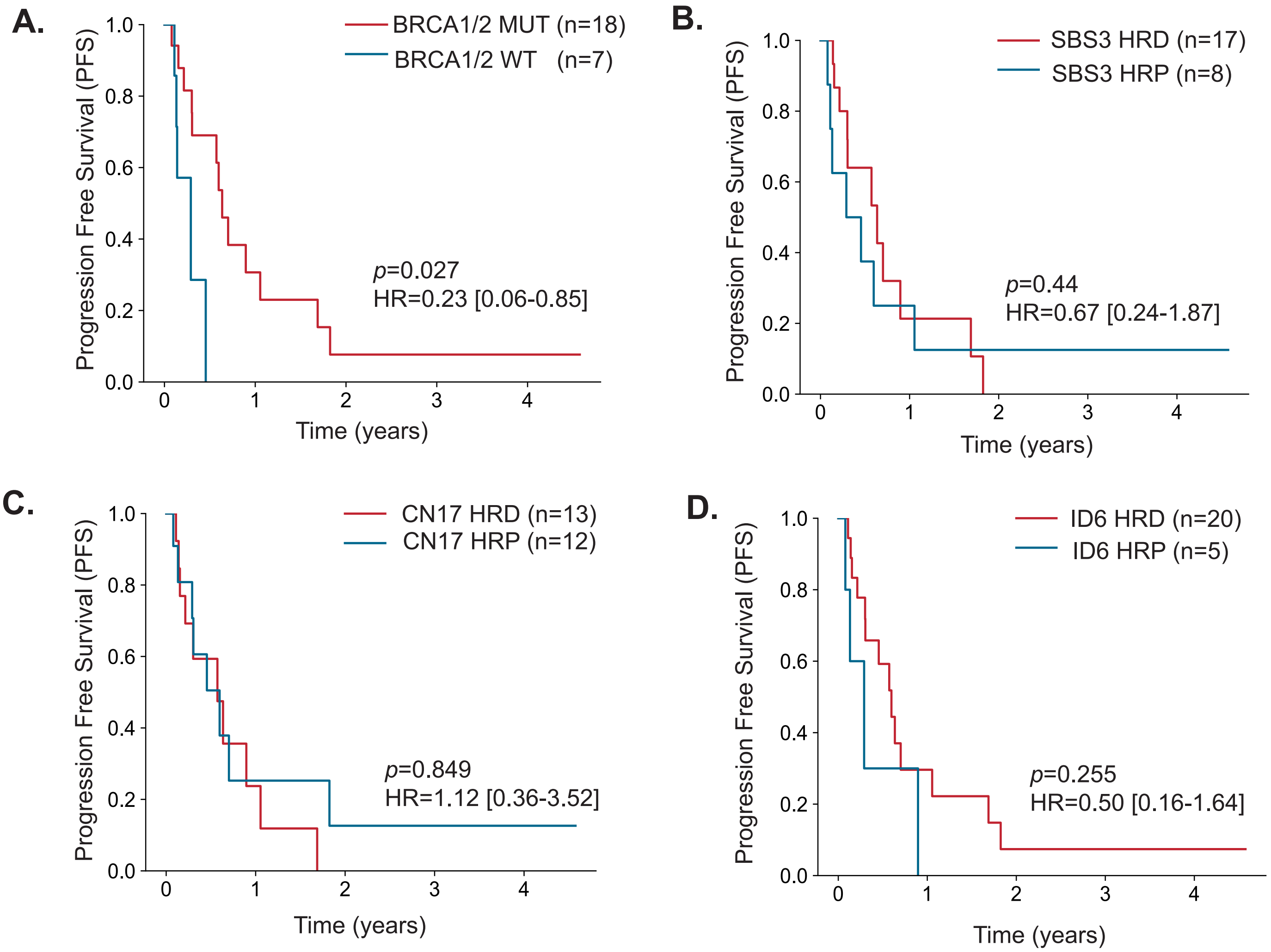

Supplementary Figure S12: Evaluating the presence of defects in *BRCA1/2* or HRD-associated signatures for predicting survival in PARP inhibitor treated ovarian cancers. Progression free survival (PFS) across 25 PARPi treated ovarian cancers stratified based on presence of ***(A)*** BRCA1/2 mutations, ***(B)*** SBS3, ***(C)*** CN17, or ***(D)*** ID6.Listed p-values and hazard ratios (HRs) are based on a Cox proportional hazards model after adjusting for age at diagnosis and tumor stage. 95% confidence intervals are provided for all HRs within the Kaplan-Meier plots.
