# Supplementary material for: HRProfiler Detects Homologous Recombination Deficiency in Breast and Ovarian Cancers Using Whole-Genome and Whole-Exome Sequencing Data
Source: Cancer Res. 2025 May 6;85(13):2504–13. doi: 10.1158/0008-5472.CAN-24-2639 (PMC12214882; doi:10.1158/0008-5472.CAN-24-2639)
Supplement: Supplementary Figure S13 — evaluates the prediction of survival in platinum-treated TCGA ovarian samples across HRD tools, the presence of defects in BRCA1/2, and HRD-associated signatures. [file can-24-2639_supplementary_figure_s13_suppsf13.pdf]

Supplementary Figure S13

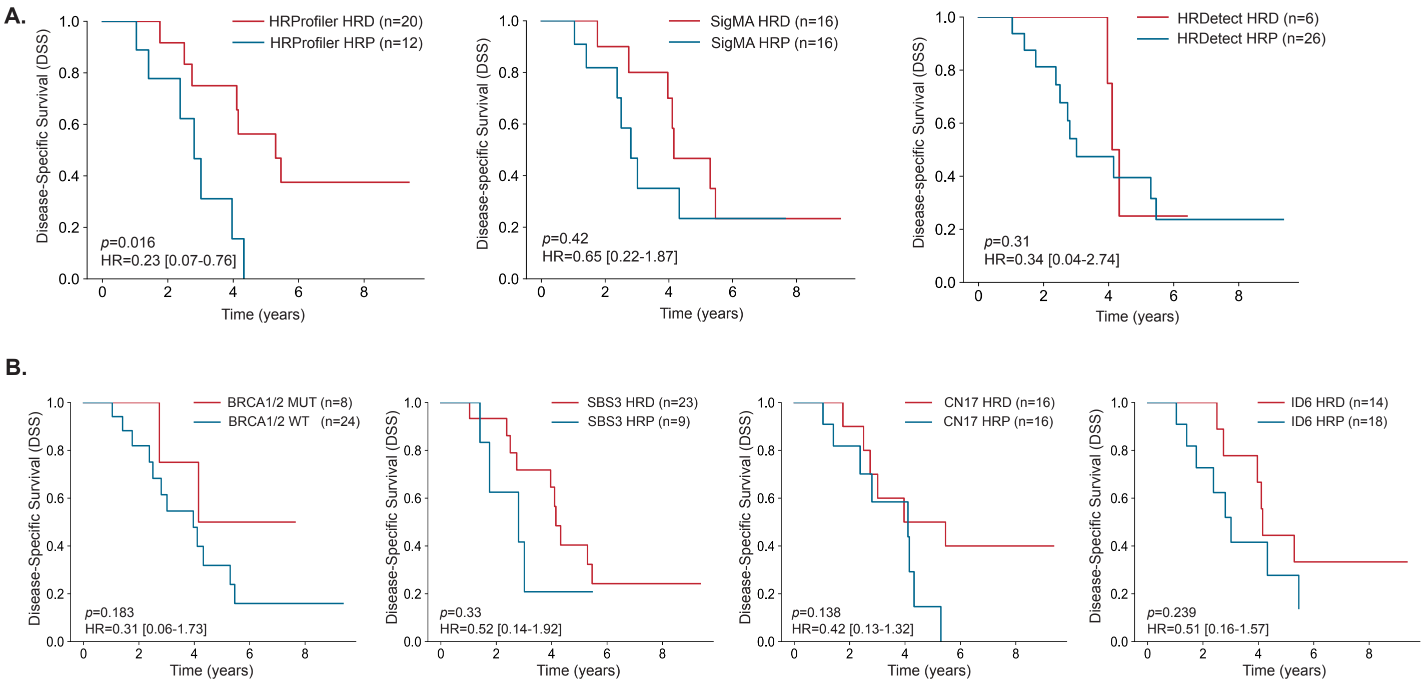

Supplementary Figure S13: Evaluating the prediction of survival in platinum-treated held-out TCGA ovarian samples across HRD tools, the presence of defects in BRCA1/2, and HRD-associated signatures. Kaplan-Meier curves for disease-specific survival (DSS) in 32 platinum-treated patients with high-grade serous ovarian cancer. Patients are classified as HRD or HRP based on predictions from: ***(A)*** HRProfiler, SigMA, and HRDetect, and ***(B)*** BRCA1/2 status and the presence of SBS3, CN17, and ID16. Listed p-values and hazard ratios (HRs) are based on a Cox proportional hazards model after correcting for age at diagnosis and tumor stage. 95% confidence intervals are provided for all HRs within the Kaplan-Meier plots.
